# Acne Management in an Immunosuppressed Renal Transplant Patient: A Case Report

**DOI:** 10.7759/cureus.109500

**Published:** 2026-05-23

**Authors:** Luis Velázquez Arenas, Sarahi Garay Enriquez, Daniela Gómez Guerra

**Affiliations:** 1 Dermatology, School of Medicine and Health Sciences at Tecnológico de Monterrey, Monterrey, MEX; 2 Medicine, School of Medicine and Health Sciences at Tecnológico de Monterrey, Monterrey, MEX

**Keywords:** 1726 nm laser, acne, dermatology, kidney transplant, laser therapy

## Abstract

Acne vulgaris is a chronic inflammatory disorder of the pilosebaceous unit with multiple therapeutic options; however, management may be challenging in patients in whom systemic therapies are not advisable or are preferably avoided due to underlying clinical conditions. Here, we report a case of a 27-year-old male with a history of kidney transplantation on chronic immunosuppressive therapy who presented with persistent moderate inflammatory acne. To avoid systemic therapy, treatment was performed using a 1726-nm sebum-selective laser. This case represents the first reported use of this technology in Mexico and highlights its potential as a safe and effective therapeutic alternative in complex clinical scenarios.

## Introduction

Acne vulgaris is one of the most common dermatologic conditions, affecting up to 85% of adolescents, although it may present across a wide age spectrum and can persist into adulthood. It is a chronic inflammatory disorder involving the pilosebaceous unit and is influenced by several risk factors, including increasing age during adolescence, a positive family history of acne, and oily skin type. Current therapeutic strategies encompass a broad range of options, including topical agents, systemic antibiotics, hormonal therapies, oral isotretinoin, procedural and device-based treatments, as well as alternative medicine and dietary and environmental interventions. More recently, a 1726-nm sebum-selective laser received clearance from the U.S. Food and Drug Administration in 2022 as a novel device-based modality for the treatment of acne [1].

The development of this technology is based on the principle of selective photothermolysis, first described by Drs. Anderson and Parrish in 1983, which enables targeted thermal injury of specific tissue structures through the use of carefully chosen wavelengths [2,3]. Supporting this concept, Sakamoto and colleagues demonstrated that optical pulses within the 1700-1720 nm range can selectively destroy sebaceous glands in ex vivo human facial skin with minimal injury to adjacent cutaneous structures [4].

The 1726-nm sebum-selective laser has been newly introduced in Mexico as a treatment option for acne vulgaris. To our knowledge, this represents the first reported case in Mexico of its use in a patient with moderate inflammatory acne and a history of kidney transplantation receiving chronic immunosuppressive therapy. This case highlights the potential role of this modality as a therapeutic alternative in complex clinical scenarios in which conventional systemic treatments may be undesirable.

## Case presentation

**Figure 1:**
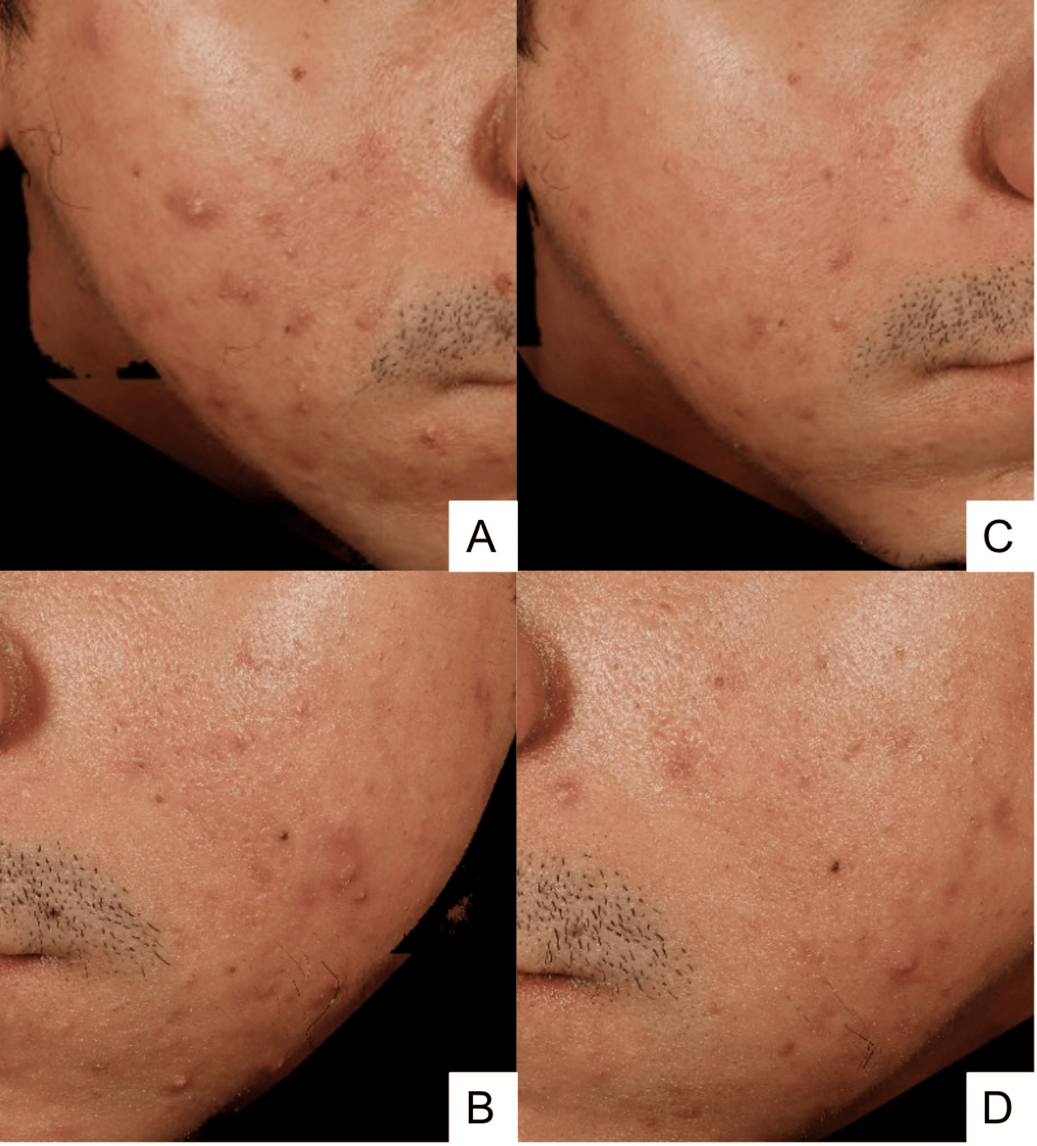
Clinical photographs of the patient. (A, B) Baseline images obtained in September 2025, prior to initiation of treatment. (C, D) Images acquired one month after completion of the four treatment sessions.

A 27-year-old male with a history of kidney transplantation performed two years prior, secondary to idiopathic renal failure, presented with persistent inflammatory acne predominantly affecting the cheeks. The patient remained on chronic immunosuppressive therapy; therefore, to avoid systemic treatment with either oral antibiotics or retinoids, due to patient preference and multidisciplinary team recommendations, alternative therapeutic options were pursued. Given the additional advantage of sustained improvement and long-term efficacy, treatment with a 1726-nm sebum-selective laser was offered and selected as the therapy of choice. The initial treatment session was performed in September 2025, following standardized clinical photography. Subcutaneous injectable local anesthesia was administered, and laser application was limited to the cheeks for assessment of procedural tolerability. A second session was conducted in November 2025 after application of a topical anesthetic formulation containing lidocaine, prilocaine, and tetracaine under occlusion for one hour; at that time, the treatment area was expanded to include the entire face, and subsequent sessions were performed using a full-face approach. The third session took place in December 2025, during which the patient requested injectable, rather than topical anesthesia. A fourth and final session was completed in January 2026; at that visit, the patient again declined topical anesthesia in favor of injectable anesthesia and reported having self-administered oral paracetamol combined with tramadol prior to arrival. Treatment parameters were adjusted according to anatomic location, with a target peak epidermal temperature (PET) of 41°C for the forehead and 43°C for the cheeks and nose; the remaining session-specific parameters are detailed in Table 1. Standard post-procedural care included strict photoprotection with daily application of broad-spectrum sunscreen. Following completion of the laser treatment course, the patient was maintained on a topical regimen consisting of a cleanser, topical clindamycin, and a fixed combination of adapalene with benzoyl peroxide applied once weekly at night, in addition to continued daily sunscreen use. The procedures were well tolerated overall. No complications or adverse events were observed throughout the treatment course, other than transient procedural pain during the sessions, with mild erythema and edema noted after laser application. At one-month follow-up, the patient demonstrated a marked reduction in inflammatory lesion count and decreased sebaceous activity, without evidence of infectious complications or delayed wound healing (Figure 1).

**Table 1:** Session-wise treatment parameters for the 1726-nm laser. PET: peak epidermal temperature.

Session number	Session time	Modes used	Laser emissions	Average PET(s)
#1	45 minutes	Standard, boost	160	42.0°
#2	62 minutes	Standard, boost	200	41.5°
#3	64 minutes	Standard, boost	224	41.6°
#4	74 minutes	Standard, boost	195	41.3°

## Discussion

Acne is a highly prevalent inflammatory skin disorder worldwide; however, its occurrence may be further increased in immunosuppressed patients. Mahé et al. (2006) reported that among 80 renal transplant recipients treated with sirolimus, 38 patients (45%) developed acne, with a significantly higher prevalence in males (75%) compared to females (6%). Furthermore, clinical improvement was achieved with topical therapies in 53% of patients, while systemic treatments were considered beneficial in 67% of cases [5]. These findings highlight the substantial burden of acneiform complications in immunosuppressed patients and the effectiveness of conventional acne treatments in this population.

However, several case reports have described an association between systemic isotretinoin therapy and renal adverse events, including acute interstitial nephritis, nephrotic syndrome, acute kidney injury, and hematuria with dysuria [6,7]. Given the patient’s history of renal transplantation and the concern for renal complications associated with systemic retinoid therapy, laser-based therapy was selected.

Over the past decades, laser therapies have emerged as a promising alternative approach for the treatment of acne vulgaris, particularly due to their potential to overcome several limitations and avoid adverse effects associated with conventional medical treatments [8]. More recently, the development of the 1726-nm laser specifically designed to target sebaceous glands has expanded the therapeutic landscape of acne management.

In a prospective multicenter study conducted by Goldberg et al. (2026), the efficacy of a 1726-nm sebum-selective laser for the treatment of mild to severe acne was evaluated in patients with Fitzpatrick skin phototype classification II-VI. Among participants who completed three treatment sessions and the scheduled follow-up, clinical response was observed in 79.8% at 12 weeks, increasing to 91.5% by 52 weeks. Transient erythema occurred in all patients, and mild edema was reported in 98.1%. Patient-reported satisfaction was high, with 75.3% describing themselves as “satisfied” or “extremely satisfied” at 12 weeks, rising to 83.1% at the 52-week evaluation [9].

A separate prospective study by Alexiades et al. (2023) assessed the safety profile and clinical outcomes of the 1726-nm sebum-selective laser in 104 patients with moderate to severe acne across different skin phototypes. Quantitative evaluation demonstrated a progressive decline in inflammatory lesion counts over time. At the four-week follow-up, 32.6% of participants achieved greater than a 50% reduction in active inflammatory lesions, with this proportion increasing to 79.8% at 12 weeks and 87.3% at 26 weeks [10]. These results further support the sustained therapeutic effect of this laser modality in the treatment of inflammatory acne.

## Conclusions

Overall, the 1726-nm sebum-selective laser represents a promising therapeutic option for the management of acne vulgaris, particularly in patients in whom conventional systemic therapies may be contraindicated, poorly tolerated, ineffective, or otherwise undesirable. As this technology has been newly introduced in Mexico, clinical experience remains limited. However, the findings of this case highlight the potential role of this laser as a safe alternative with favorable outcomes in selected patients with inflammatory acne, consistent with the currently available literature, and provide early clinical insight into its use in Mexico. In addition, emerging long-term data suggest sustained clinical improvement after treatment, further emphasizing the potential of this modality as a durable non-systemic option in acne management. Therefore, documenting early national experiences with this technology is essential to expand evidence regarding its efficacy, safety, and applicability in the Latin American population.
